# Development and initial evaluation of a semi-automatic approach to assess perivascular spaces on conventional magnetic resonance images

**DOI:** 10.1016/j.jneumeth.2015.09.010

**Published:** 2016-01-15

**Authors:** Xin Wang, Maria del C. Valdés Hernández, Fergus Doubal, Francesca M. Chappell, Rory J. Piper, Ian J. Deary, Joanna M. Wardlaw

**Affiliations:** a Department of Neuroimaging Sciences, Centre for Clinical Brain Sciences, University of Edinburgh, Edinburgh, UK; b Centre for Cognitive Ageing and Cognitive Epidemiology (CCACE), University of Edinburgh, Edinburgh, UK; c College of Medicine and Veterinary Medicine, University of Edinburgh, Edinburgh, UK; d Department of Psychology, University of Edinburgh, Edinburgh, UK

**Keywords:** Virchow–Robin spaces, Perivascular spaces, MRI, Small vessel disease, WMH, white matter hyperintensities, PVS, perivascular spaces, BG, basal ganglia, CS, centrum semiovale, T1W, T1-weighted MRI sequence, T2W, T2-weighted MRI sequence, PVH, periventricular white matter hyperintensities, DWMH, deep white matter hyperintensities, SVD, small vessel disease

## Abstract

**Purpose:**

Perivascular spaces (PVS) are associated with ageing, cerebral small vessel disease, inflammation and increased blood brain barrier permeability. Most studies to date use visual rating scales to assess PVS, but these are prone to observer variation.

**Methods:**

We developed a semi-automatic computational method that extracts PVS on bilateral ovoid basal ganglia (BG) regions on intensity-normalised T2-weighted magnetic resonance images. It uses Analyze™10.0 and was applied to 100 mild stroke patients’ datasets. We used linear regression to test association between BGPVS count, volume and visual rating scores; and between BGPVS count & volume, white matter hyperintensity (WMH) rating scores (periventricular: PVH; deep: DWMH) & volume, atrophy rating scores and brain volume.

**Results:**

In the 100 patients WMH ranged from 0.4 to 119 ml, and total brain tissue volume from 0.65 to 1.45 l. BGPVS volume increased with BGPVS count (67.27, 95%CI [57.93 to 76.60], *p* < 0.001). BGPVS count was positively associated with WMH visual rating (PVH: 2.20, 95%CI [1.22 to 3.18], *p* < 0.001; DWMH: 1.92, 95%CI [0.99 to 2.85], *p* < 0.001), WMH volume (0.065, 95%CI [0.034 to 0.096], *p* < 0.001), and whole brain atrophy visual rating (1.01, 95%CI [0.49 to 1.53], *p* < 0.001). BGPVS count increased as brain volume (as % of ICV) decreased (−0.33, 95%CI [−0.53 to −0.13], *p* = 0.002).

**Comparison with existing method:**

BGPVS count and volume increased with the overall increase of BGPVS visual scores (2.11, 95%CI [1.36 to 2.86] for count and 0.022, 95%CI [0.012 to 0.031] for volume, *p* < 0.001). Distributions for PVS count and visual scores were also similar.

**Conclusions:**

This semi-automatic method is applicable to clinical protocols and offers quantitative surrogates for PVS load. It shows good agreement with a visual rating scale and confirmed that BGPVS are associated with WMH and atrophy measurements.

## Highlights

• User-friendly semi-automatic perivascular spaces segmentation method is efficient in quantifying PVS from conventional T2-weighted MRI.
• Semi-automatically determined perivascular spaces count and volume agree with visual ratings.
• Semi-automatic perivascular spaces assessment proves useful for clinical studies and MRI protocols.
• Rigorous statistical analysis evaluating the new method on a longitudinal stroke sample.
• Potential for use in studies of cerebral small vessel disease and in a range of other neurological diseases.

## Graphical abstract

**Figure:**
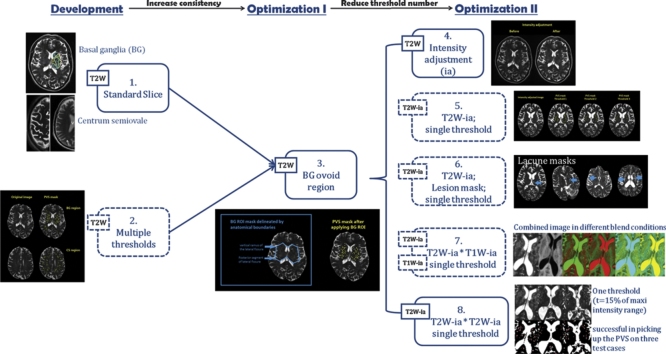

## 1 Introduction

Perivascular spaces (PVS), or Virchow–Robin spaces, have been defined as fluid-containing spaces that surround the walls of arteries, arterioles, veins and venules as they course from the subarachnoid space into the brain parenchyma (Etemadifar et al., 2011, Kwee and Kwee, 2007). PVS are round or linear delineated structures seen on MRI with intensities close to cerebrospinal fluid (CSF) and less than 3 mm (Kwee and Kwee, 2007) diameter in cross section (Wardlaw et al., 2013). PVS may function as drainage and fluid circulation pathways for soluble and insoluble material through the central nervous system (Rennels et al., 1985). They may provide imaging evidence of vascular and inflammatory changes in the brain. PVS are specific sites for immune cell accumulation, reaction and transmigration into the brain parenchyma (e.g., leukocytes, dendritic cells, T-cells, B-cells and macrophages (Polledo et al., 2012, Sagar et al., 2012, Wuerfel et al., 2008)). More PVS visible on MRI are associated with increasing age, cognitive impairment, cerebral small vessel disease (SVD) lacunar stroke and white matter hyperintensities, (WMH), multiple sclerosis, and may be related to altered blood brain barrier permeability (Doubal et al., 2010, MacLullich et al., 2004, Potter, 2011, Potter et al., 2013, Wardlaw, 2010, Zhu et al., 2010).

PVS on MRI are commonly assessed using visual rating scales, several of which have been proposed (MacLullich et al., 2004, Potter, 2011, Potter et al., 2013, Zhu et al., 2010, Chen et al., 2011, Patankar et al., 2005, Rouhl et al., 2008). These differ in how they score the anatomical location or range of PVS, as summarised in (Potter, 2011). Potter and colleagues (Potter, 2011) reviewed and evaluated the ambiguities and advantages in the existing PVS visual rating scales and combined their strengths to develop a more comprehensive visual rating scale (available at http://www.sbirc.ed.ac.uk/documents/epvs-rating-scale-user-guide.pdf). This new scale used standard T2-weighted (T2W) structural brain MRI to assess the severity of the PVS located in three major anatomical regions (midbrain, basal ganglia (BG) and centrum semiovale (CS)).

Potential confounds in PVS visual ratings have been previously discussed (Potter, 2011): differences in PVS visibility, presence of WMH, varying number of PVS on different slices, ‘double counting’ of linear PVS, poor scan quality including movement, asymmetry in background brain appearances, asymmetry in PVS, presence of focally dilated PVS that could be mistaken for lacunes, and differences between most severe categories and variations in lesion load between cohorts. Therefore, all existing rating scales share similar limitations: intra- and inter-observer differences and ceiling and floor effects caused by the few categories into which PVS are condensed. These limitations could be overcome by the use of computational methods, which, if they can avoid observer bias and provide a quantitative rather than qualitative PVS measure, may provide more precise estimates of PVS severity and also allow their size & volume to be measured. Such methods could be useful to detect more subtle differences in PVS between subjects, for example calculating the percentage of PVS volume in the total brain volume and investigating an association between PVS volume and WMH volume.

To our knowledge, few studies have described any computational methods suitable for PVS quantification (Wuerfel et al., 2008, Descombes et al., 2004, Uchiyama et al., 2008). A systematic review found 6 studies that used computational methods to assess PVS and 4 studies that presented approaches with potential for quantifying PVS (Valdes Hernandez et al., 2013). Though these computational methods were promising, they have not been widely used in the target population of patients with small vessel disease, and required to be validated on scans acquired with different protocols.

In this paper, we present a user-friendly computational method for counting the number and measuring the volume of PVS in relevant regions-of-interest (ROI) as a surrogate for PVS load to be used in large clinical research studies. We present the development of the method, results from calculating the intra- and inter-observer agreement in a small number of representative test cases, describe thresholds that work in most cases, and describe the methods for assessing difficult cases. Then we evaluate the method's performance in 100 patients by comparing the PVS computational counts and volumes with the validated visual rating scale scores. Finally, we investigated the associations between BG PVS count & volume, and WMH rating scores (periventricular hyperintensities: PVH; deep white matter hyperintensities: DWMH) and volume, atrophy rating scores and brain volume.

## 2 Materials and methods

### 2.1 Sample selection

For developing and testing the computational approach, the imaging datasets for 16 subjects were chosen from a sample of older subjects: The Lothian Birth Cohort 1936 Study (http://www.lothianbirthcohort.ed.ac.uk/) (LBC1936) to represent a full range of PVS, WMH, lacunes, and brain atrophy based on previous analyses (Potter, 2011, Deary et al., 2007, Deary et al., 2012). The MRI protocol has been previously published (Wardlaw et al., 2011).

For evaluation, we applied the method to 100 patients with clinical features of a lacunar or mild cortical stroke recruited in a study of stroke mechanisms: 100 had full baseline imaging assessments and 46 returned for follow-up imaging after a median of 39 months (IQR 30–45 months). Full study details have been published previously (Wardlaw et al., 2009).

### 2.2 Brain MRI acquisition

All MRI acquisition was conducted in the Brain Research Imaging Centre, University of Edinburgh (http://www.bric.ed.ac.uk). A GE Signa Horizon HDx 1.5T clinical scanner (General Electric, Milwaukee, WI), equipped with a self-shielding gradient set and manufacturer-supplied eight-channel phased-array head coil, was used to acquire T2W, T2*- and T1-weighted (T2*W, T1W) and Fluid Attenuation Inversion Recovery (FLAIR) datasets amongst other sequences. Full details of the MR protocol for both studies are described elsewhere (Doubal et al., 2010, Wardlaw et al., 2009, Wardlaw et al., 2011). The characteristics of the sequences relevant for assessing PVS are summarised in Table 1.

### 2.3 Brain MRI acquisition

#### 2.3.1 Pre-processing

The main sequence used to identify PVS was T2W (Kwee and Kwee, 2007, Wardlaw et al., 2013, Doubal et al., 2010, Potter, 2011, Potter et al., 2013). T1W and FLAIR images were also used as references to differentiate PVS from other lesions such as WMH, infarcts and lacunes (Groeschel et al., 2006). T1W and FLAIR, were rigidly registered to the corresponding T2W volumes using FLIRT (FMRIB Linear Registration Tool) (Jenkinson et al., 2002) from the FMRIB Software Library (Oxford, UK), (www.fmrib.ox.ac.uk/fsl). The intracranial volume (ICV) was extracted from the T2*W sequence, using the Object Extraction Tool in Analyze™ 10.0 (Analyze Direct, Inc. Overland Park, KS, USA). False positives (i.e., non-ICV structures like the pituitary gland, clivus and sphenoid sinus) were removed manually (Valdes Hernandez et al., 2012).

#### 2.3.2 Development and optimisation of the multi-stage segmentation method

**Fig. 1:**
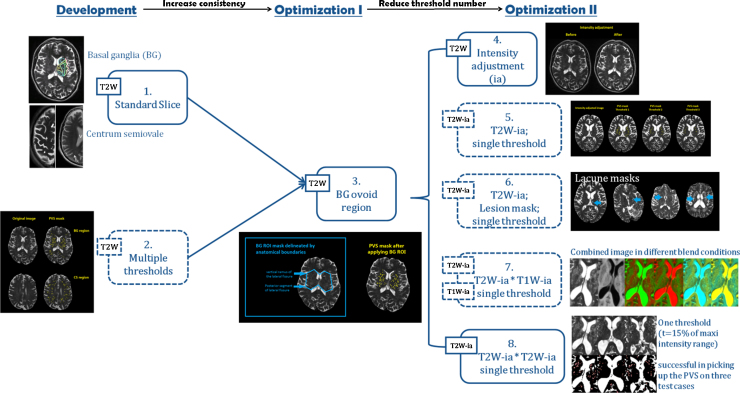
Steps in a multi-stage PVS segmentation approach. T2W-ia: T2-weighted image after intensity adjustment; T1W-ia: T1-weighted image after intensity adjustment. The solid lines are the steps included in the final procedure for the PVS segmentation method: selection of the “target” slice (1), delineation of the “ovoid” region of interest (3), adjust T2W image's intensity (4), and combine two intensity-adjusted T2W images (8). Dotted lines are steps tested in the development stage but not included in the final procedure: apply multiple thresholds (2), apply directly a single threshold to the intensity adjusted T2W images (5), exclude (i.e., mask) lesions with similar intensities: e.g., lacunes and stroke lesions (6), and combine T1W with T2W after intensity adjustment (7).

We used the module ‘Object counter’ in Analyze 10.0™ (http://analyzedirect.com/documents/training_guide/measure/Object_Counter.pdf) to develop our segmentation technique. Fig. 1 schematically represents all the steps that we applied during method development and optimisation: the steps with solid outline were ultimately included in the final procedure for general PVS segmentation.

**Fig. 2:**
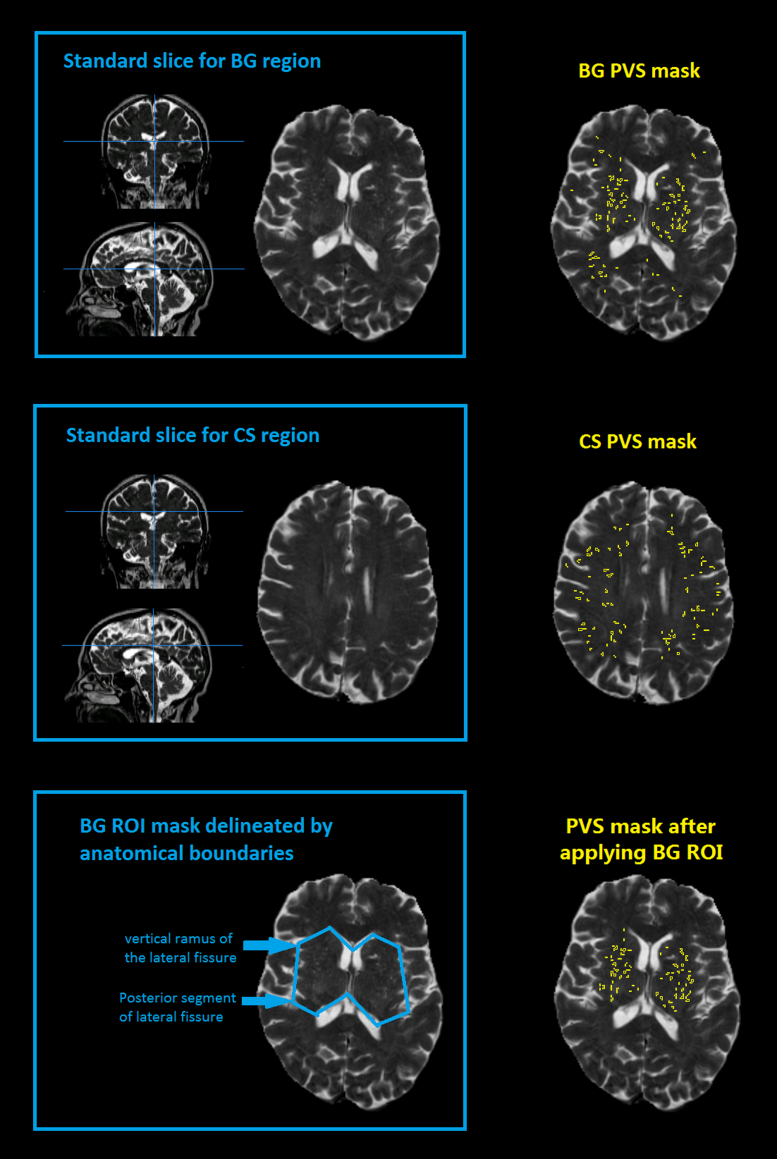
Standard slices for the basal ganglia (BG) and centrum semiovale regions, and regions of interest (ROI) specifically to the BG regions delineated by anatomical boundaries.

PVS are commonly seen in the BG and CS (Kwee and Kwee, 2007) as the arterioles in these regions extend over a relatively long distance in straight lines and can be followed in or through the plane of scan. Based on a carefully developed and evaluated clinical PVS visual rating scale (Potter, 2011), we selected a representative axial slice in the BG and CS. In the BG, we chose the slice that contains at least one characteristic BG structure (i.e., caudate nucleus, internal capsule, thalamus, lentiform nucleus, external capsule and insular cortex) and also shows the most PVS. In the CS, we chose a slice between the superior aspect of the bodies of the lateral ventricles and the subcortical white matter near the vertex (see Fig. 2). Tests of intra- and inter-observer reliability (both visual rating and computational identification) showed high variability in the identification of the elongated PVS that are common in the CS and outer regions of the BG slice. Therefore, we restricted our assessment to bilateral ovoid regions on the BG slice, one in each hemisphere, delineated by the vertical ramus of the lateral fissures and the posterior segment of the lateral fissures as shown in Fig. 2.

Analysis of the intensity distribution in the T2W images revealed that PVS have intensity levels ranging from 30% to 90% of the maximum signal intensity on this MRI sequence that vary even within the same subject, presumably due to factors such as partial volume averaging (Valdes Hernandez et al., 2013), making it unfeasible to assess PVS using a single threshold without any intensity adjustment. Therefore we performed a linear intensity adjustment in 3 stages: normalisation, gamma correction and linear mapping. The general equation for the gamma correction is:

(1) $${i^{\prime }}_{\left(x,y,z\right)}=I_{\max}\times {\left(\frac{\left(i_{\left(x,y,z\right)}-I_{\min}\right)}{\left(I_{\max}-I_{\min}\right)}\right)}^{\left(\text{gamma}+I_{\text{min}}\right)}$$

where *I*_max_ and *I*_min_ are the maximum and minimum intensity levels and *i′*_(*x,y,z*)_ and *i*_(*x,y,z*)_ are the resultant and initial intensity levels for a voxel identified by the coordinates (*x,y,z*). Initially we used a linear intensity transformation (gamma equal 1) saturating the 1% of the lowest and highest intensities at the minimum and maximum values, respectively. However, it was necessary to transform the intensity levels quadratically to successfully apply one threshold on each of the test cases. This was achieved doing a voxelwise product of the intensity-adjusted-T2W image with itself, or, as *I*_min_ was zero, using a gamma correction factor of 2.

We performed additional tests to try to improve PVS segmentation such as combining T1W and T2W images and manually excluding lacunes (Wardlaw et al., 2013, Valdes Hernandez et al., 2013) (see Fig. 1, steps outlined by dotted lines), but neither step improved the results. We identified a general pattern that involved the use of only three thresholds, each one to be applied according to the T2W image characteristics of the individual patient as follows:

(1) Low Threshold (7.5% of maximum signal intensity) was suitable for a patient that had scattered PVS, a uniform, visually normal intensity background, and few focal lesions such as WMH or lacunes (1/16 case).
(2) High Threshold (15% or above of maximum signal intensity) was suitable for patients that either had grouped PVS, or a high background signal, or many other lesions (WMH, lacunes, mineral deposits), or with poor scan quality, for example from patient movement (8/16 cases).
(3) Medium Threshold (11.25% of maximum signal intensity) was suitable for patients that did not have the characteristics requiring use of either low or high thresholds (7/16 cases).

During development, measurements were done by two observers (Ob1, Ob2) on two separate occasions. Ob1 used the same standard slices at both times. Ob2 was blinded to the slice chosen by Ob1 for a first measurement, and was unblinded for a second measurement. Ob1 was an experienced image analyst, while Ob2 had about half as much image analysis training. Bland-Altman plots (Bland and Altman, 1986) were used to estimate the agreement within or between observers. The BG PVS intra-observer agreement for PVS count was: mean difference = 5.00, SD = 32.80, and PVS volume (voxels): mean difference = −5.37, SD = 57.20. When both observers were blind to the slice selection, the BG inter-observer agreement for PVS count was: mean difference = 23.60, SD = 46.10; and PVS volume (voxels): mean difference = 80.30, SD = 134.70. When both observers selected the same slice, the agreement was far much better: for PVS count it was mean difference = 0.50, SD = 4.87; and PVS volume (voxels): mean difference = 4.69, SD = 29.95, being these only caused by differences in the threshold selection.

The final procedure for general PVS segmentation was determined to be:

(1) Choose the standard slice in the BG region.
(2) Apply intensity adjustment to the T2W image.
(3) Combine two T2W intensity adjusted images.
(4) Set the region of interests into two BG ovoid regions (BG ROI).
(5) Adjust interactively the maximum and minimum size of the ‘hyperintense’ objects as per Valdes Hernandez et al. (2013)
(6) Apply one threshold (Low, Medium or High) according to the image characteristics. T1W and FLAIR images would be used as reference image.
(7) For difficult and complicated cases, another threshold or the same threshold plus manual editing should be applied.

For most cases, the final PVS segmentation would take 8 to 13 min per case in total, and for complicated cases, it might need an additional 5 to 20 min including the application of multiple thresholds or further manual editing.

### 2.4 Testing in 100 subjects with mild stroke

To evaluate the new PVS segmentation method, we applied the final procedure as above for general PVS segmentation to 100 patients with ischaemic stroke, all of whom had imaging at presentation with stroke and recruitment into the primary study, 46 of whom also had follow-up scanning about three years later. Visual rating of PVS, WMH and brain atrophy was available for all of these cases at both time points.

A neuroradiologist rated the PVS in the BG and CS regions as follows: 0 = no PVS, 1 ≤ 10 PVS, 2 = 10–20 PVS, 3 = 21–39 PVS, and 4 ≥ 40 PVS, separately in the left and right hemispheres, and an ‘overall’ score for both hemispheres, using a scale previously developed by our group (Doubal et al., 2010, MacLullich et al., 2004, Potter, 2011, Potter et al., 2013). White matter hyperintensities (WMH) were coded using Fazekas scores, for periventricular (PVH) and deep lesions (DWMH) separately in the left and right hemispheres and a combined score for both hemispheres (Wardlaw et al., 2011). Brain atrophy were coded using a validated template (Farrell et al., 2009), with superficial and deep atrophy coded separately ranging from none to severe in a scale from 1 to 6 according to the centiles into which the template is divided, being 1(<25th), 2(25–50th), 3(50–75th), 4(75–95th), 5(>95th) and if ≫5, 6 is used. Total atrophy was calculated as the average of deep and superficial atrophy scores.

CSF and WMH volumes (ml) were measured using validated semiautomatic brain tissue segmentation software (Valdes Hernandez et al., 2010) (http://sourceforge.net/projects/bric1936/). Brain volume was obtained by subtracting CSF from ICV. Full image segmentation details have been published previously (Wang et al., 2012).

### 2.5 Statistical analysis

We used linear regression to test association between BG PVS count, total PVS volume and PVS visual rating scores. We also explored the association between BG PVS count and individual PVS volume. In the 100 stroke patients, we also used linear regression to investigate the association between BG PVS count & volume, WMH rating scores (periventricular: PVH; deep: DWMH) and volume, atrophy rating scores and brain volume. All linear regression analyses were performed in R version 2.15.2 (http://www.r-project.org/).

## 3 Results

The following results refer to the 100 patients with mild stroke, 51 of whom had lacunar stroke and 49 who had cortical stroke. The mean age was 69 years (range 37–92 years). The median stroke severity score with the National Institute for Health Stroke Scale was 2, 62% had hypertension and 15% had diabetes. WMH volumes ranged from 0.4 to 119 ml (median 11 ml, IQR 21.7 ml), and total brain tissue volume from 0.65 to 1.45 l (median 1.14 l, IQR 0.13 l). Full details are published elsewhere (Wardlaw et al., 2009).

### 3.1 Comparison between BG PVS computational results (count & volume) and PVS visual rating scores

**Fig. 3:**
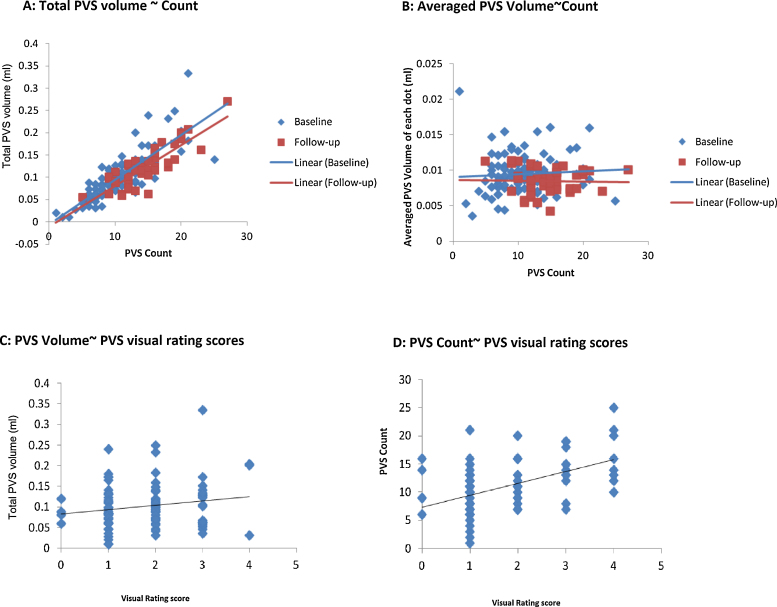
Association between computational PVS volume and count and visual rating scores in both baseline and follow-up scans in 100 patients with mild stroke. (A) Total PVS volume vs. PVS count. The *x* axis represents the PVS count, i.e., number of PVS dots obtained from the PVS computational method and the *y* axis represents the total PVS volume expressed in millilitres. The blue diamonds are results from 100 patients at baseline. The red squares are follow-up results from 46 of the 100 patients who had scans at both time points. (B) Averaged PVS individual volume vs. count in both baseline and follow-up (note association almost null at baseline and null at follow-up). (C) PVS computational total volume vs. visual rating score. (D) PVS computational count vs. visual rating score.

In the baseline 100 cases, the median of BG PVS count was 10 and the median of BG PVS total volume was 0.093 ml. The BG PVS total volume increased significantly with BG PVS count [coefficient of linear regression: 0.11; 95% confident intervals (95%CI): 0.09 to 0.12, *p* < 0.001], which means that an increase of 1 BG PVS “dot” represents an increase of 0.11 ml in the BG PVS volume (Fig. 3A). There was no association between the average volume per patient of individual PVS (here expressed as average PVS volume per count) and the PVS count (Fig. 3B), which is consistent with the visual observation that in general most PVS on MRI have similar sizes, whether few or many in number.

BG PVS count increased with overall BG PVS visual scores (2.11, 95%CI 1.36 to 2.86, *p* < 0.001). BG PVS total volume also increased with overall BG PVS visual scores (0.022, 95%CI 0.012 to 0.031, *p* < 0.001) (Fig. 3C and D). There was little association between BG PVS count & volume and CS PVS visual rating scores (Table 2).

**Fig. 4:**
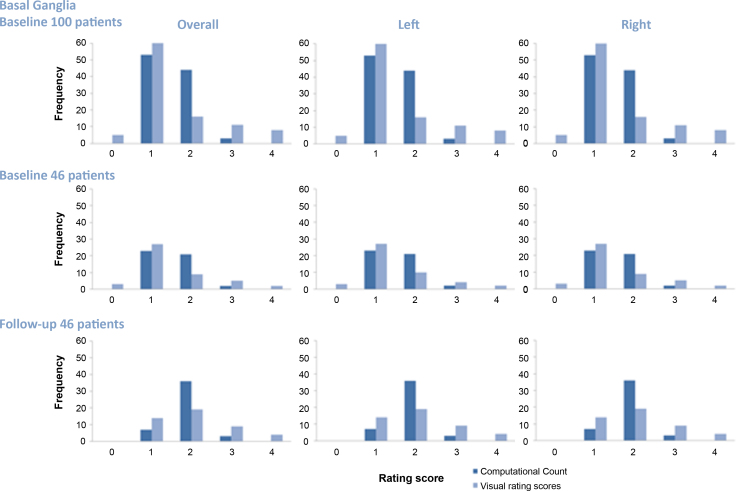
Comparison of PVS computational count condensed into a ‘score’ of similar range to the visual rating categories and PVS visual rating scores at baseline and follow-up. The *x*-axis in the histograms represents the values of the score intervals. The *y*-axis represents the frequency of two variables’ occurrence. The results of the condensed score from PVS computational count are labelled in dark blue and the results from the PVS visual rating scores are labelled in light blue.

We condensed the raw PVS computational count with the same categories as the PVS visual rating scale: from 0 to 4 (score 0 = none PVS dots; 1 = 1–10 PVS dots; 2 = 11–20 PVS dots; 3 = 21–40 PVS dots; 4 ≥ 40 PVS dots or with high background intensity). We found a similar spread of values for PVS computational count and visual count scores (similar distributions, Fig. 4). However, the PVS visual rating score classed some cases as 0 or 4 whereas the computational count had no patients in these classes, implying that the visual rating overlooks one or two PVS (classing them as ‘0’) and classes high counts as ‘4′ reflecting that the visual rating is an estimate of PVS number not a precise count. The PVS computational count always put more patients in category 2.

Additionally, the histograms (Fig. 4) illustrate several points: (a) the computational and visual counts have a very similar distribution with the median score 1 and with the second commonest score 2; (b) the difference between the ratings increases as the score increases indicating that it is harder to quantify larger numbers of PVS; (c) the visual rating scores tend to measure less than the PVS computational count when there are few PVS, and more when there are large numbers of PVS. In the 46 patients with baseline and follow-up scans obtained on average 3 years later, there were more PVS on the follow-up scans (median score 2), indicating that PVS count increases as SVD progressed (Fig. 4).

### 3.2 Association between BG PVS computational count & volume and WMH rating scores & volume

**Fig. 5:**
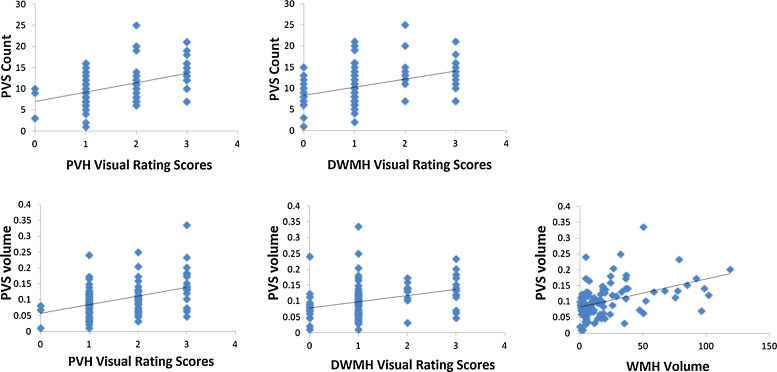
PVS count & volume (ml) vs. WMH (PVH, DWMH Fazekas visual rating scores, and volume).

BG PVS computational count was positively associated with WMH visual rating both on baseline and follow-up scans (baseline 100 patients, overall PVH: 2.20, 95%CI 1.22 to 3.20, *p* < 0.001; overall DWMH: 1.92, 95%CI 0.99 to 2.85, *p* < 0.001). BG PVS count was also positively, although weakly, associated with WMH volume (ml) (baseline 100 patients, 0.065, 95% CI 0.034 to 0.096, *p* < 0.001) (Fig. 5, Table 3).

### 3.3 Association between PVS computational count & volume and cerebral atrophy score/brain volume

**Fig. 6:**
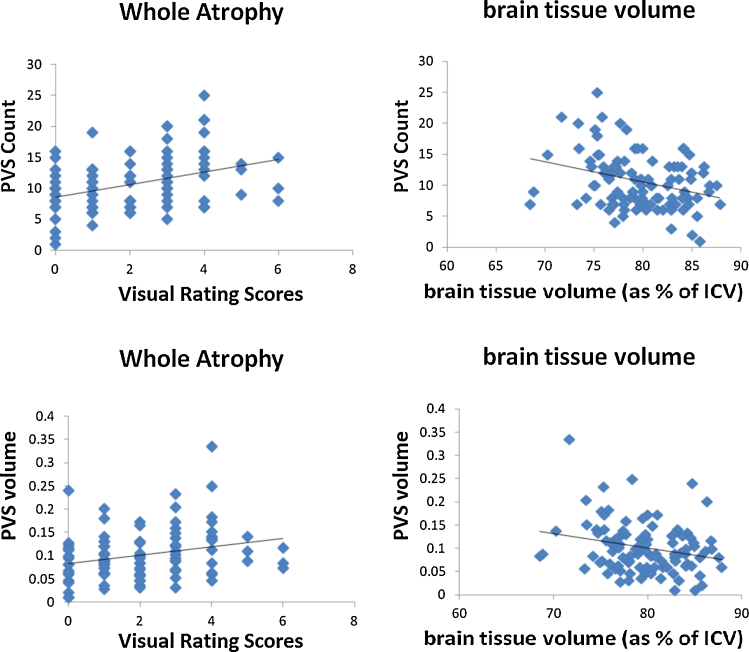
PVS count & volume (ml) vs. atrophy (brain atrophy visual rating scores, and brain tissue volume expressed as a percentage of ICV).

BG PVS count was positively associated with whole brain atrophy visual rating (defined as per Section 2.4) (1.012, 95%CI 0.489 to 1.535, *p* < 0.001). BG PVS count also increased as brain volume (expressed as the percentage of ICV) decreased (−0.326, 95%CI −0.526 to −0.127, *p* = 0.002) (Fig. 6). BG PVS total volume showed similar associations (Table 4).

### 3.4 Interrelationship of the associations at baseline and follow-up

In the subgroup of 46 patients with scans at baseline and long term follow-up, we found the same associations as for the 100 patients at baseline, both at baseline and follow-up 3 years later. The only difference was that the number of PVS increased (see above; data available on request).

## 4 Discussion

This semi-automatic intensity threshold-based computational PVS quantification method shows promise for assessing BG PVS count and volume from conventional clinical MR scans, and could complement or even replace existing visual rating scales. As PVS count is performed automatically rather than visually, it complements the subjective visual rating scales by achieving a precise quantification (i.e., count and volume) of the PVS on the BG region though increases the assessment time (as visual methods are very quick). Despite a full characterisation of the PVS (e.g., whole-brain assessment, regional density, individual delineation) could not be achieved, this study demonstrates the pursuit of this, one that can be learned from and built upon, undoubtedly constituting a step forward on the study of this imaging biomarker.

### 4.1 Method development

We used blinded observer reliability analyses and the best available knowledge from visual PVS rating methods to develop a method to computationally assess PVS using a conventional structural T2W MRI sequence. We considered 3D analysis, multi-sequence approach, and finally tested the PVS assessment in two brain regions (BG and CS) before deciding to focus only on the BG. The slice thickness of the T2W (5 mm), base sequence to identify PVS (Kwee and Kwee, 2007, Wardlaw et al., 2013, Doubal et al., 2010, Potter, 2011, Potter et al., 2013, Valdes Hernandez et al., 2013), did not allow a 3D analysis and conspired against the reliability on identifying PVS that run longitudinally, which were affected by partial volume effects. In addition, the CS region was frequently affected by a high background signal caused by diffuse WMH, limiting PVS detection. Therefore, to guarantee reliability on our measurements, we needed to restrict our analysis to only two axial ROIs at the BG level where the vessels run transversally (i.e., from the base of the brain upwards) and were less affected by the presence of diffuse WMH. The results from the inter-observer reliability analysis (both count and volume) suggest that the agreement between observers could be affected by the extent of PVS when these coalesce with white matter hyperintensities, but it's hard to make a definite statement given the small sample size.

Gamma correction affects the intensity distribution. We tested multiple gamma values and chose the best one that proved optimum for the two MRI protocols evaluated. This method can deal with difficult and complicated cases with high background signal or diffuse WMH by increasing the number of threshold steps and performing manual removal of false positives.

### 4.2 Associations of PVS computational count & volume with PVS visual rating scores, WMH and atrophy

The present study evaluated associations between PVS computational count, total PVS volume, and PVS visual rating scores, in 100 patients with mild stroke, showing significant association between PVS volume and PVS count in the BG region. However, the average PVS size (PVS volume per count) was not associated with the PVS count, consistent with visual observations that PVS size in general does not vary with number. The PVS computational results (PVS count and volume) also agreed well with a widely used PVS visual rating score in the BG.

When the BG PVS count was converted into the same categories as used in the visual rating score and compared with the BG PVS visual rating scores, the results showed a similar pattern with a similar spread of curves, interval range and peaks. However, visual rating scores showed wider dynamic range than the computational count converted to scores. The BG PVS count ‘scores’ (i.e., condensed from PVS count) and PVS visual rating scores had very good agreement when PVS were few (<20) but the agreement decreased with increasing number of PVS: similar to the inter-observer agreement problem for PVS visual ratings (Potter, 2011). To overcome this difficulty, visual rating scores assign highest score (e.g., 4 in Potter, 2011) to cases with high background signal or diffuse WMH.

Previous studies have shown a positive association between visually rated BG and CS PVS scores (Doubal et al., 2010, Potter, 2011). However, we found no association between the BG PVS volume, count, and the CS visual rating scores in this study (Table 2), perhaps due to differences of PVS CS volume and count between observers.

From the analyses performed, we found that the visual rating score categories could benefit from further modifications. For example, the category intervals are equal in size from score 0 to 2 (0 = no PVS, 1 ≤ 10 PVS, 2 = 10–20 PVS), but not at higher scores (e.g., for score 3 it is 21–40 PVS). Score 3 could possibly be narrowed down (e.g., 21–30 PVS) to spread scores better and avoid the ceiling effect.

### 4.3 Strengths

The main strengths of the current study are the rigorous and meticulous development and evaluation of the PVS measurement method in the 16 test cases and the validation in clinical scans from 100 cases, 46 of whom were followed-up three years later to test the effect of disease progression. This study gives detailed evidence of the caveats on the computational assessment of PVS on clinical scans, constituting a step forward towards the fully characterisation of this imaging biomarker.

We assessed both PVS count and volume following clinically validated criteria (Potter, 2011, Valdes Hernandez et al., 2013). The associations seen for the PVS computational method (volume and count) are consistent with the associations between PVS visual rating scores and WMH seen in previous work (Doubal et al., 2010, Zhu et al., 2010, Rouhl et al., 2008). The computational method shows less ceiling and floor effects, therefore would be useful in studies which quantify few or many PVS, providing reliable quantitative information that constitutes an adequate surrogate of PVS load on older individuals and small vessel disease sufferers.

### 4.4 Limitations

The limitations of the method are that it is not fully automated and requires manual intervention. However, almost no image analysis software are available for analysing brains of patients with stroke that do not require some manual intervention. The efficiency in picking up PVS requires some observer knowledge and skill and can be impeded by poor quality images. The final optimised method only focused on the PVS in the BG region. A whole brain 3D computational assessment would have allowed to analyse PVS spatial distribution, orientation and regional PVS density among other parameters, but this only would have been possible with high resolution and high quality isotropic multimodal structural MRI, which require long scanning times not tolerated by most stroke patients.

The fact that this method is not suitable for reliably assessing PVS in the CS could be considered a limitation, especially because PVS in the BG and CS may not represent the same pathology or be associated with the same risk factors. However, studies have demonstrated that only using the BG PVS load (represented by the visual rating scores) in addition to the burden of microbleeds, WMH and asymptomatic lacunar infarcts, is it possible to estimate a total score representative of the total burden of cerebral SVD, which has been associated with general cognitive ability in older age (Staals, 2015) and decreased cognitive function in lacunar stroke and hypertensive patients (Huijts et al., 2013). Moreover, PVS in the BG and not in CS have been associated with lacunar stroke subtype whilst CS PVS scores have not been reported to differ between stroke subtypes (Doubal et al., 2010), thus reinforcing the usefulness of this method for the study of SVD.

### 4.5 Future directions

The method should be tested with more observers and on other scanning protocols at different field strengths, as this study involved only two observers and images from only 16 older community-dwelling subjects and 100 patients with stroke obtained at 1.5T. Since this method does not include intense programming and is user friendly, it could be easily used for further PVS assessment on other clinical studies. Though the present study quantifies PVS in patients with lacunar or cortical strokes, literature also suggested PVS may relate to other manifestations of cerebral small vessel disease (SVD) and cognitive dysfunction (Wardlaw, 2010) therefore being a potential imaging marker for SVD and cognitive dysfunction (MacLullich et al., 2004, Potter et al., 2013, Zhu et al., 2010). PVS are also seen in MS patients (Etemadifar et al., 2011, Wuerfel et al., 2008) and are a marker of inflammation (Aribisala et al., 2014, Rouhl et al., 2010, Rouhl et al., 2012, Satizabal et al., 2013). This computational method could potentially be used in further studies of SVD and cognition, such as in studies of lacunar infarcts and WMH (Rouhl et al., 2008), blood-brain barrier dysfunction (Wardlaw et al., 2009), vascular dementia, Alzheimer's disease or mild cognitive impairment (Chen et al., 2011), atrophy. PVS also appear on MRI scans from young and healthy volunteers who participate as controls for studying the mechanisms of ageing and brain diseases, and so this method could also be applied to scans from normal younger individuals (Aribisala et al., 2014).

Further improvements of the current method are necessary. We presented its development using an interactive semi-automated tool, but it can be streamlined and fully automated, although a final visual check and manual removal of false positives may be required afterwards. If the whole BG is automatically extracted, depending on the slice thickness, a full 3D characterisation of the BG and midbrain PVS could be achievable.

## Conflict of interest statement

The authors disclose no potential conflicts of interest.

## Figures and Tables

**Table 1:** Characteristics of the MRI sequences used in this study.

Study/parameters	Mild stroke study (Doubal et al., 2010, Wardlaw et al., 2009)	LBC1936 study (Wardlaw et al., 2011)
TR/TE/TI (ms) T1W	9.7/3.984/500	9.7/3.984/500
TR/TE (ms) T2W	6300/107	11320/102
TR/TE (ms) T2*W	620/15	940/15
TR/TE/TI (ms) FLAIR	9000/140/2200	9000/140/2200
Pixel bandwidth (kHz)	15.63 (T1W, FLAIR) 25 (T2W) 12.5 (T2*W)	15.63 (T1W, FLAIR) 20.83 (T2W) 12.5 (T2*W)
Matrix	256 × 216 (T1W) 384 × 224 (T2W, T2*W) 384 × 224 (FLAIR)	192 × 192 (T1W) 256 × 192 (T2W, T2*W) 256 × 256 (FLAIR)
No. slices	256 (T1W) 28 (T2W, T2*W) 28 (FLAIR)	160 (T1W) 80 (T2W, T2*W) 40 (FLAIR)
Slice thickness (mm)	1.02 (T1W) 5 (T2W, T2*W) 5 (FLAIR)	1.3 (T1W) 2 (T2W, T2*W) 4 (FLAIR)
Inter-slice gap (mm)	1	0
Voxel size (mm^3^)	1.02 × 0.9 × 1.02 (T1W) 0.47 × 0.47 × 6 (T2W, T2*W) 0.47 × 0.47 × 6 (FLAIR)	1.3 × 1.3 × 1 (T1W) 1 × 1 × 2 (T2W, T2*W) 1 × 1 × 4 (FLAIR)

Legend: ms: millisecond; KHz: kilohertz; mm: millimetre; mm^3^: cubic millimetre; FLAIR: fluid attenuated inversion recovery; T1W: T1-weighted MRI sequence; T2W: T2-weighted MRI sequence; TR: repetition time; TE: echo time; TI: inversion time.

**Table 2:** Associations between PVS computational count & volume (ml), and PVS visual rating scores in basal ganglia (BG) and centrum semiovale (CS) regions of baseline scans in 100 patients with mild stroke.

Association (PVS computational count & volume ∼ predictor)	Coefficient of change in PVS per unit change in predictor	95% confidence intervals	*p* Value
PVS computational count ∼ PVS volume	67.3	(57.9, 76.6)	<0.001
PVS computational count ∼ PVS visual rating scores BG	2.11	(1.36, 2.86)	<0.001
PVS computational count ∼ PVS visual rating scores CS	0.677	(−0.279, 1.633)	0.16
PVS volume ∼ PVS visual rating scores BG	0.022	(0.012, 0.031)	<0.001
PVS volume ∼ PVS visual rating scores CS	0.010	(−0.001, 0.022)	0.081

**Table 3:** Associations between PVS computational count & volume (ml), and WMH Visual Rating Scores (i.e., PVH and DWMH Fazekas scores) of baseline scans in 100 patients with mild stroke.

Association (PVS computational count & volume ∼ predictor)	Coefficient of change in PVS per unit change in predictor	95% confidence intervals	*p* Value
PVS computational count ∼ WMH visual rating scores (Fazekas total)	0.065	(0.034, 0.096)	<0.001
PVS computational count ∼ WMH visual rating scores PVH	2.199	(1.215, 3.182)	<0.001
PVS computational count ∼ WMH visual rating scores DWMH	1.919	(0.990, 2.848)	<0.001
PVS volume ∼ WMH visual rating scores (Fazekas total)	8.895E − 04	(5.235E − 04, 1.256E − 03)	<0.001
PVS volume ∼ WMH visual rating scores PVH	0.027	(0.015, 0.039)	<0.001
PVS volume ∼ WMH visual rating scores DWMH	0.019	(0.008, 0.031)	0.001

**Table 4:** Associations between PVS computational count & volume (ml), and brain atrophy visual rating scale (deep and superficial) of baseline scans in 100 patients with mild stroke.

Association (PVS ∼ predictor)	Coefficient of change in PVS per unit change in predictor	95% confidence intervals	*p* Value
PVS computational count ∼ Atrophy visual rating scores overall	1.012	(0.489, 1.535)	<0.001
PVS computational count ∼ Atrophy visual rating scores deep	1.614	(0.732, 2.495)	<0.001
PVS computational count ∼ Atrophy visual rating scores superficial	1.774	(0.701, 2.846)	0.001
PVS computational count ∼ brain volume (% in ICV)	−0.326	(−0.526, −0.127)	0.002
PVS volume ∼ Atrophy visual rating scores overall	0.009	(0.002, 0.016)	0.009
PVS volume ∼ Atrophy visual rating scores deep	0.016	(0.005, 0.027)	0.005
PVS volume ∼ Atrophy visual rating scores superficial	0.013	(0.000, 0.027)	0.055
PVS volume ∼ brain volume (% in ICV)	−0.003	(−0.006, −0.001)	0.013
